# Lectures and Observations on Clinical Surgery

**Published:** 1847-01

**Authors:** 


ART. XIV.—*Lectures and Observations on Clinical Surgery.* By ANDREW ELLIS, F.R.C.S.I. &c.—*Dublin*, 1846. 8vo, pp. 275.

THIS volume contains the substance of certain clinical lectures delivered by the author at the Jervis-street Hospital and the Cecilia-street School of Medicine, in Dublin, and was published, he adds, at the expressed desire of some of the pupils who attended the hospital during the session.

The subjects treated of are medical education, wounds of arteries, traumatic aneurisms, injuries of the head and their consequences, peritonitis and wounds of the abdomen, delirium tremens, catalepsy and hydrophobia. These topics are handled with a respectable degree of ability, and though we have looked in vain through the Lectures for anything which can entitle them to be considered as a valuable addition to medical literature, they nevertheless contain directions as to the details of practice such as form the proper staple of a clinical lecture, and which, no doubt, rendered them useful to those to whom they were originally addressed.

Whether such a publication was needed by the "students of Great Britain and Ireland," to whom the author has somewhat ambitiously addressed it, is another matter, and one to which we should be disposed to give a pretty decided negative. We cannot refrain, moreover, from adding that the author's literary researches have proved singularly infelicitous since they led him to the discovery of only *scattered* informaticn in the works of former writers on such subjects as those we have named above. We think Mr. Ellis would have better consulted his own interests and that of his readers in offering his lectures to some one of our Journals than by publishing them in their present form; at all events we will venture to suggest for the future, seeing that his book is now *un fait accompli*, that he would act more wisely before committing himself to the public to consult some judicious senior on the matter, rather than to act on suggestions of admiring pupils.

We observe in his lecture on hydrophobia that the author not only comes to the conclusion that the bite of a dog, not rabid, can produce hydrophobia in the human species, but expresses his belief that the saliva of a dog in perfect health, and in a state of tranquillity when it neither bites nor attempts to bite, may, if applied to a wound, produce hydrophobia in the human subject. This, to say the least, is somewhat strong doctrine, and by no means sufficiently borne out by the evidence adduced, which consists of two or three cases related at second-hand, and without any of those details necessary to satisfy the mind as to the absence of fallacies so likely to exist in a matter of this sort. On this point we beg to refer our readers to an article on the work of Dr. Wright, in the preceding part of the present Number.

